# Removing a Cystein Group On Interferon Alpha 2b at Position 2 and 99 does Not Diminish Antitumor Activity of the Protein, Even Better

**DOI:** 10.3797/scipharm.ISP.2015.04

**Published:** 2016-02-14

**Authors:** Heni Rachmawati, Adhitya Jessica, Yeyet Cahyati Sumirtaputra, Debbie Sofie Retnoningrum, Amirah Adlia, Ratih Asmana Ningrum

**Affiliations:** 1 School of Pharmacy, Bandung Institute of Technology, Ganesha 10, 40132, Bandung, Indonesia; 2 Department of Pharmacokinetics and Drug Delivery, University of Groningen, Antonius Deusinglaan I, Groningen, Netherlands; 3 Research Center for Biotechnology, Indonesian Institute of Sciences, Jalan Raya Bogor KM 46, 16911, Cibinong, Indonesia

**Keywords:** Interferon alpha 2b, Antiproliferation, Apoptosis, p21k1, p27, Caspase 3, pSTAT1, Flow cytometri, Mutein

## Abstract

Interferon alpha 2b is the only standard therapeutic protein for hepatitis virus infections. Further study demonstrated that this protein also posseses antitumor activity in several cancerous organs. One main pathway of this antitumor activity is mediated through antiproliferation as well as proapoptotic effects. Previously, we have successfully developed recombinant human interferon alpha 2b (rhIFNα2b) by using a synthetic gene. In addition, two mutein forms of rhIFNα2b were generated to improve the characteristics of this protein. Two point mutations showed better pharmacokinetic profiles than one point mutation as well as the native form. In the present study, this mutein form was studied for ist antitumor effect *in vitro* using HepG2 cells. As a comparison, the native form as well as a commercial rIFNα2b were used. Several parameters were investigated including the MTT assay, cell viability test, cell cycle using flow cytometric analysis, and the genes and protein expressions involved in cell growth. The latest was observed to study the mechanism of rhIFNα2b. There was no significant difference in the MTT assay and cell viability after cells were treated with both forms of rhIFNα2b. However, the mutein rhIFNα2b tended to show better proapoptotic activity reflected by flow cytometric data, protein expression of pSTAT1, and DNA expression of caspase 3.

## Introduction

Interferons are a group of multi-functional cytokines that were originally identified as the proteins responsible for the formation of cellular resistance to bacterial lipopolysaccharides (LPS) and viral infections [1]. Interferons also play a role in the control of cell growth, differentiation and regulation of the immune system. Therefore, their used in anticancer therapy had been well documented for a long time and often possess relatively high antitumor activity. Among them the leucocytic interferon α-2b is the most studied and is successfully used for therapy of various cancers such as skin and ocular melanoma, renal, bladder, ovarian and breast cancer, Kaposhi’s sarcoma, myeloma, chronic lympholeukosis etc [2]. The effect of interferon alpha 2b as anticancer is mediated through either antiproliferative effect or proapoptotic mechanism or combination of them. Interferon affects different phases of the mitotic cycle in different cell systems with the most common effect is G1 arrest [3]. As reported by Asano et al, IFN-α affected cell cycle arrest of mouse macrophages showed that the Cdk inhibitors p19 and p21 were strongly up-regulated after treatment with IFN-α, and that the binding of these inhibitors to the G1 cyclin/Cdk complex leads to reduction of its kinase activities and results in G1 arrest in the early phases of IFN treatment [4]. Treatment with interferon induces Cdk inhibitors p15 and p27 [5–7] as well, resulting in cell-cycle arrest at the G1 phase.

We have successfully overproduced recombinant human interferon alpha 2b generated from synthetic gene-encoded human interferon alpha 2b [8]. In addition, 2 mutein forms were established by substituting cystein groups at position 2 and 99 with aspartic acid [9]. Substitution was done through site directed mutagenesis. The main aim of this modification was to prolong the biological half life of the protein. This was proven by our study on the pharmacokinetic profile of the mutein forms after intravenous administration in animal model. As reported, the mutein forms especially 2 points mutation (C2D C99D) showed longer plasma half life as compared to native protein [9].

In present report, we described the biological activity of our developed human recombinant 2 points mutation of interferon alpha 2b (C2D C99D) and then compared to native form. A commercial interferon alpha 2b was also used. We focused our study on the antiproliferation effect *in vitro* using HepG2 cells. The MTT assay was used for the standard antiproliferation assay. Cell counting was done for MTT assay confirmation. Several genes involved in the cell cycle were evaluated to study the pathway undergone by interferon alpha 2b for the antiproliferation effect. In addition, proapoptotic effect of our interferon alpha 2b was also studied through cell cycle analysis using flow cytometry and analysis of pSTAT1 expression at protein level by Western Blot. Since STAT1 has been shown to promote apoptosis and carry tumor suppressor functions in different types of cancers, the correlation among parameters we reported here will help to explain the mechanism of our developed interferon alpha 2b in the cancer treatment.

## Results and Discussion

### Results

**Fig. 1:**
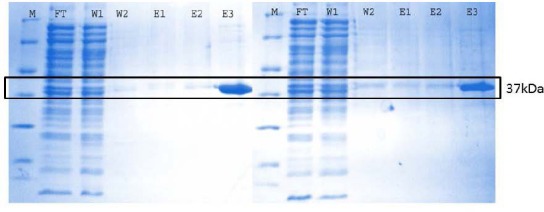
SDS PAGE analysis of native (A) and 2 points mutein (B) IFNα2bs. (M = protein marker, FT = *flow* through, W = *washing*, E = *eluate*)

The expression of purified both native and 2 points mutant of rIFNα2bs on SDS PAGE is depicted in figure 1 as a single band with molecular weight of ~ 37 kDa. As the substitution was only involved 2 cystein residues (242 Da) replaced with 2 aspartic acid residues (266 Da), no different protein product was clearly appeared.

**Fig. 2:**
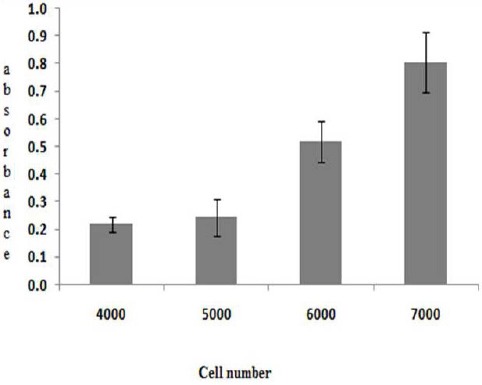
The influence of cell number on MTT assay response. Optimum cell number was 6000 cells/well.

**Fig. 3:**
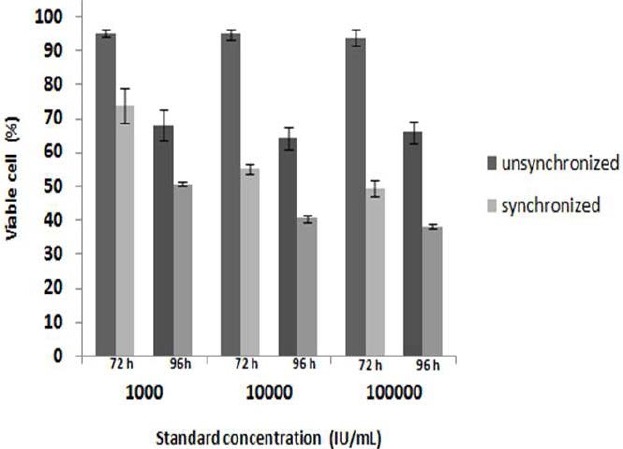
The influence of pretreatment and incubation time on the cell viability. Optimum cell response was achieved by synchronization on pre treatment by exposing cell 18 h to free serum medium

Figure 2 describes the optimization of cell number on MTT assay, showing that the number of HepG2 cells was in line with the UV/Vis absorbance of formazan. By considering the highest absorbance is not more than 0.8, 6000 cells were used for further studies. Cell synchronization in free serum medium for 18 hours showed better result comparing to untreated cells. Optimization of incubation time resulted in better growth inhibitory effect at 96 hours (Fig 3). However due to the limitation of working time of the antibiotics (three days at 37 °C), 72 hours incubation time was used for next study.

**Fig. 4:**
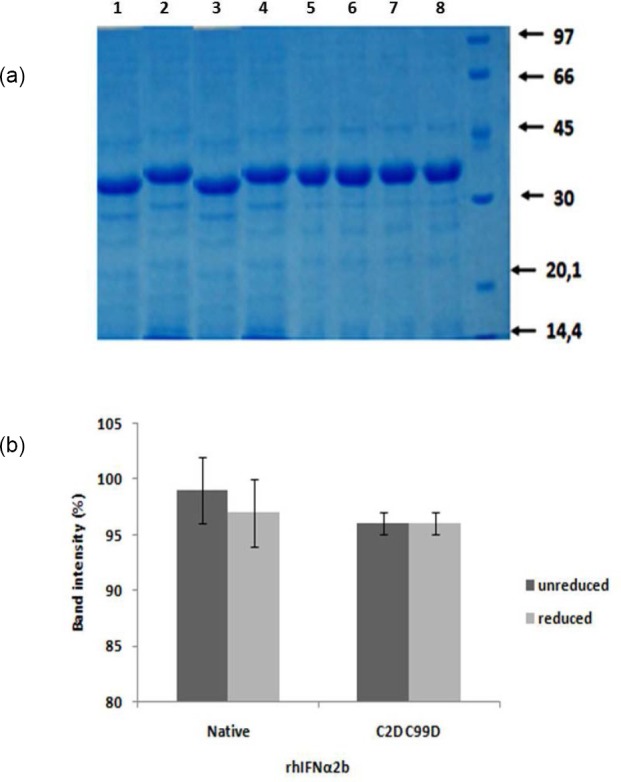
Protein stability monitoring at optimum MTT assay condition: A. Electrophoregram profile of non-reducing SDS PAGE, lane 1= unreduced native control, 2= reduced native control, 3 = unreduced native at optimum condition, 4= reduced native at optimum condition, lane 5= unreduced mutein control, 6= reduced mutein control, 7 = unreduced mutein at optimum condition, 8 = reduced mutein at optimum condition

Monitoring of the stability of the proteins at optimum conditions of MTT assay showed similar electrophoregram profile with the control (rhIFNα2b native and mutein form stored at 4°C). The stability of protein can be monitored through different migration due to the number of disulfide bonds and protein degradation. The stability of mutein form can only be observed through the presence of degradant bands as the number of existing disulfide bonds did not provide different migration (Fig 4A). The analysis of bands intensity using ImageJ software showed that all proteins were stable indicating by >97% of the band intensity as compared to the control (Fig 4B).

**Fig. 5:**
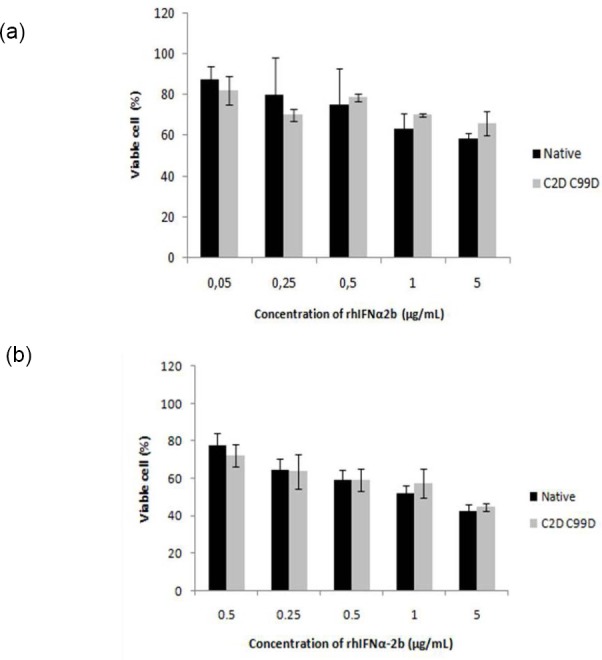
The dose-dependent profile of antiproliferation activity of native and 2 points mutein form of rhIFNα2b: A. MTT assay B. Manual cell counting by tryphan blue staining. There was no significant difference after cells were treated with both forms of rhIFNα2b.

As seen in figure 5A, the optimum concentration of protein showing highest cell growth inhibitory effect was 5 µg/mL. This MTT assay result was in line with tryphan blue staining (Fig 5B). It is suggested that both native and 2 point mutein of rhIFNα2bs showed similar antiproliferation potency. The substitution of cystein with aspartate did not alter the antiproliferative activity of rhIFNα2b.

**Fig. 6:**
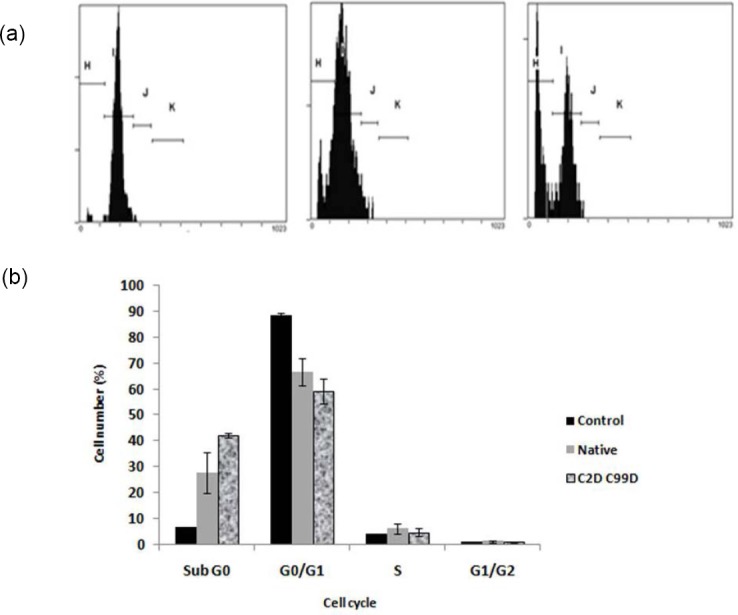
The influence of treatment on the HepG2 cell cycle. A. Flow cytometric analysis ilustrating celullar DNA content. DNA histogram number 1= control, 2= native rIFNα2b, 3= mutein rIFNα2b (C2D C99D); B. The percentage of cell number in different phase of cycles. Protein concentration was 5 ug/mL and treatment time was 72 h. The mutein rhIFNα2b tends to show better proapoptotic activity.

Figure 6 presents the cell division phase determined using flowcytometry. As seen, both forms of rhIFNα2b have stronger ability to induce apoptosis over to the control. DNA histogram with propidium iodide staining indicated that mutein form produced more apoptotic cells than the native (Fig 6A).

**Fig. 7:**
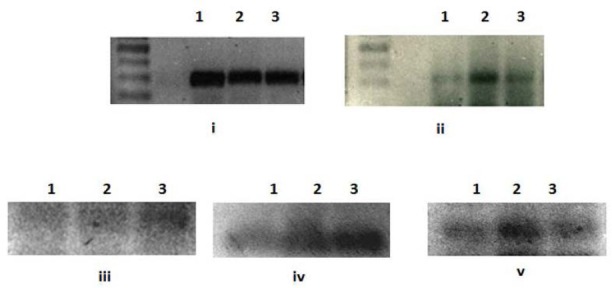
Molecular weight analysis of various genes expressed after rIFNα2bs treatment on agarose gel. 1= control, 2= native rIFNα2b, 3= mutein rIFNα2b; i to v in sequence: GAPDH, p27kip1, caspase-3, p21V1, p21B.

Antiproliferation and proapoptotic effects of rhIFNα2b were further confirmed by the analysis of gene expression. cDNA amplification products were shown in figure 7. Semi-quantitative analysis of PCR product using ImageJ indicated that the expression of several genes increased (Table 3). The expression was corrected with GAPDH as a house keeping gene.

**Tab. 1:**
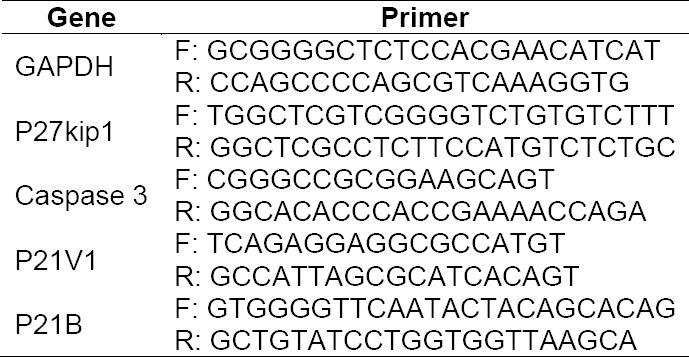
Nucleotide sequence of primers used to express genes involved in the antiproliferation effect of both native and muteins rIFNα2bs

**Tab. 2:**
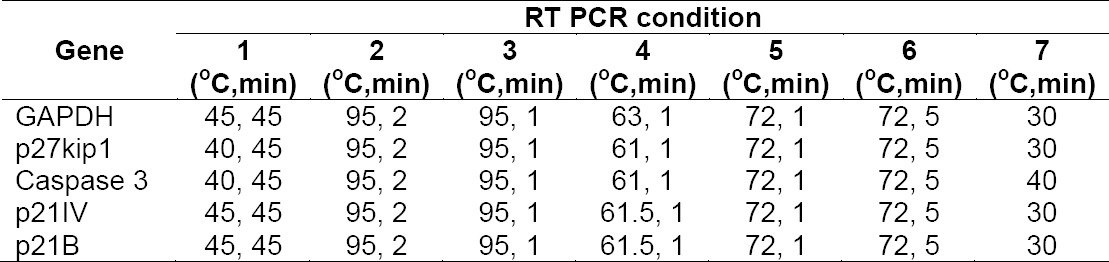
Amplification conditions of targeted genes from HepG2 treated cells

**Tab. 3:**
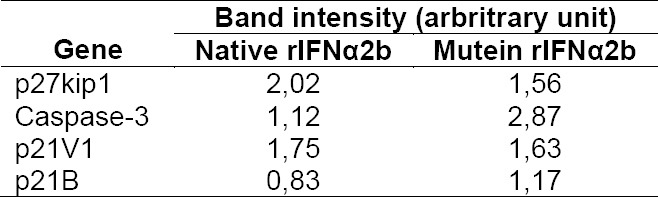
Semi quantitative analysis of genes expressed after induction with rIFNα2bs

**Fig. 8:**
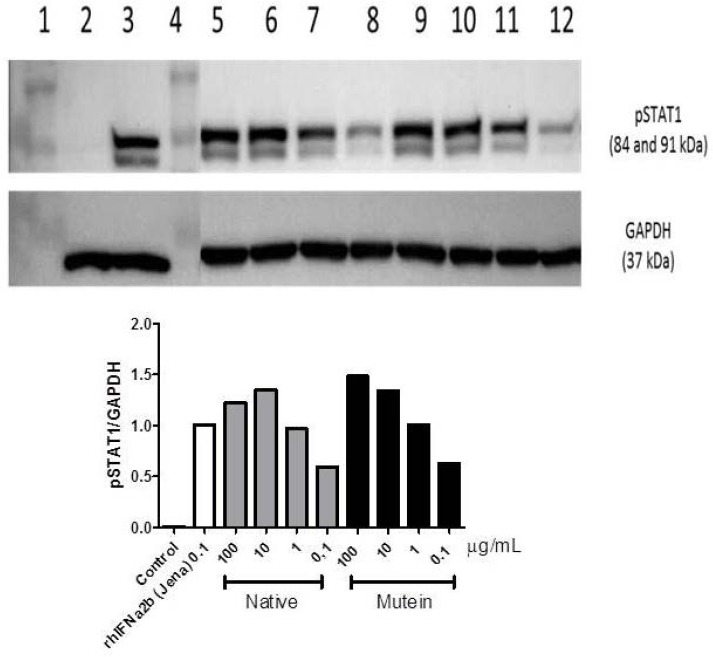
pSTAT1 expression after cells were treated with interferon alpha 2b. (A) Western Blot analysis of pSTAT1: 1= protein marker, 2 = untreated cells, 3 = treated with commercial IFN-α2b, 5-8 = treated with native IFN-α2b (100 µg/mL, 10 µg/mL, 1 µg/mL, 0.1 µg/mL), 9-12 = treated with mutein rIFN-α2b (100 µg/mL, 10 µg/mL, 1 µg/mL, 0.1 µg/mL). (B) Semiquantitative analysis of pSTAT1 expression.

The expression of protein level of pSTAT1 (phosphorylation of STAT1, Fig 8) confirmed the gene expression of caspase-3 indicating that proapoptotic effect of mutein rIFN-α2b is indeed more prominent as compared to native form with concentration dependence. The activation of STAT1 by the interferon receptor complex is responsible for the transcription of a significant portion of IFN induced genes. Many of these genes are responsible for the induction of an apoptotic state in response to IFN as also reported in our study.

## Discussion

The correct rIFNα2bs both native and mutein were confirmed by SDS PAGE shown in figure 1. The stability of these forms during experiment is important to confirm the activity and revealed in figure 4. The main aim of our work is to investigate whether our established native rIFNα2b as well as modified form demonstrate activity in cell proliferation. HepG2 cells were used as a cell line model based on our previous study (data not published) which expresses IFN-alpha receptor. The IFN alpha receptor expression is very important requirement to mediate biological activity of this cytokine. In addition to classical assay for anti proliferation effect (MTT and cell counting), we also investigated the potency of our rIFNα2bs to induce cell apoptosis, as well as to upregulate the genes and protein involved in the cell cycle. The genes we reported here are p27kip1, caspase-3, p21VI, and p21B. Experimental optimizations were performed to obtained appropriate condition to convince our data concerning the effect of our developed rIFNα2b and its modification on proliferation inhibition.

Our rIFNα2bs demonstrated stimulatory effect on all genes studied which are responsible for supression of cell growth (figure 7). This indicates that removing cystein residue at position 2 and 99 did not diminish the interferon biological activity, even better stimulating effect in some genes (caspase-3 and p21IV).

IFN is the only growth inhibitory cytokine that has a wide clinical use. With regard to the mechanism of IFNs antiproliferative activity we reported here and in line with the study of Matsuoka et al, rIFNα2b has mainly been performed in established cell lines [10–12]. In those studies, it was demonstrated that one of the first molecular effects on the cell cycle machinery following the addition of IFN is an upregulation of p21 levels, that is independent of functional p53 [10]. This upregulation of p21 seems to trigger secondary events such as inhibition of Cdks and upregulation of p27 [14]. Furthermore, the rapid increase in p21 expression seems to be a common denominator of many cell lines that become cell cycle arrested by IFN-a [13–16].

As shown in flow cytometric data (Fig 6), other prominent activity of Interferons (IFNs) is their ability to induce cell cycle arrest. This activity has then been proposed to be of main pathway in mediating the antitumor effect of them. In several IFNR expressed cell lines, an immediate upregulation of the cyclin dependent kinase inhibitor p21 occurs following IFN-alpha treatment. Thi is thought to play a major role as an effector for this phenomenon by triggering further events. Most of the proteins responsible in controlling the cell cycle have been designated as products of tumor suppressor genes due to the association of mutations of these genes with different types of cancers.

The enzymes that regulate cell cycle progression, the cyclin-dependent kinases (CDKs) are candidates to integrate growth control signals with the cell cycle machinery. These enzymes, which are composed of a catalytic kinase subunit and a regulatory subunit called cyclin, are regulated by multiple mechanisms, including the rate of synthesis, subcellular localization and degradation rate of the cyclin subunit. CDK activity is also regulated by stimulatory and inhibitory phosphorylation events as well as by the binding of cyclin-dependent kinase inhibitors. There are two families of CKIs, the Cip/Kip family and the InK4 family. The Cip/Kip family consists of p21, p27, and p57 and is characterized by a conserved NH_2_-terminal CDK-binding domain, exclusive binding to heterodimeric complexes and affinity for multiple cyclin/CDK complexes.

As confirmed by other study, treatment of HepG2 cells with both our native and mutein forms of rIFNα2b resulted in increased expression of cyclin-dependent kinase inhibitors p21 and p27. Because p21 and p27 CDK activity, they are suggested the principal mediator of rIFNα2b as antiproliferative protein.

In mammalian cells, proliferation control is primarily achieved in the G1-phase of the cell cycle [17-20]. After G1, cells become largely independent of extracellular signals and progress automatically through subsequent cell cycle phases to the next G1 [18]. Hence, the G1 CDKs are likely to play a particularly important role in the integration of growth control signals with the cell cycle machinery.

The CKI p21 [21–23] may be a critical regulator of CDK activity. In normal cells, a significant fraction of cyclin/CDK complexes are found in quaternary complexes associated with p21 and PCNA [24]. Kinase activity of cyclin/CDK complexes may be dependent on p21 stoichiometry such that inhibition by p21 may require the association of more than one molecule of p21 per complex [25]. A p21 role in CDK regulation may be important in the regulation of the normal cell cycle.

In particular apoptotic induction, mutein form shows better activity than the native form. Better activity of 2 points mutation confirms the prolonged half life as reported previously by Ningrum et al [9]. Explanation can be proposed due to consistent conformation of 2-point mutein form of rIFNα2b after substituting 2 cystein groups hence removing the disulphide bond with aspartic residue.

The effect of interferon alpha to induce apoptosis is well documented. Its action in the apoptosis pathways is also as a possible anti-tumour mechanism [26]. Several studies have shown that IFN can exert direct cytotoxic effects on primary malignant cells and tumour cell lines *in vivo* [27–29] and IFN has also been demonstrated to be a direct inducer of apoptosis [29, 30]. Induction of apoptosis is thus a highly attractive mechanism of action for IFN’s anti-tumoral response and it could also play a role in the clearing of virus infected cells.

Two major cell-intrinsic pathways have been described for induction of apoptosis, one beginning at the level of cell surface death receptors, the other involving activation of mitochondria followed by release of cytochrome *c*. The induction of these pathways triggers the activation of caspase cascades [31, 32]. To date, 14 different caspases have been described [33, 34]. Although overexpression of each of these can kill cells by apoptosis, not all of them are normally involved in this process. Caspases-3, -6 and -7 are the major effector caspases and when activated they cleave the vast majority of proteins that undergo proteolysis in apoptotic cells [33, 35, 36]. The upregulation of caspase 3 (Fig 7) described here after treatment cells with our rIFNα2b is confirmed the antiproliferation effect of interferon alpha 2b observed with MTT assay (Fig 5A) and cell counting (Fig 5B). In line with gene expression responsible for proapoptotic effect of this rIFNα2b, the protein level of pSTAT1 was also upregulated in treated cells with native and mutein rIFNα2b, in dose-dependent manner (Figure 8). As also confirmed by others [37], the antitumor activity of interferon alpha 2b is mediated by a combination of proliferation inhibition and apoptotic induction. Taken together, it is suggested that the mechanism of interferon alpha 2b we developed in the controlling cell growth is via regulation of cell cycle and controlling the apoptosis process. Caspase-3 which was induced by pSTAT1 seems to be playing important role in this effect. Two cystein groups of recombinant interferon alpha 2b did not contribute to the anticancer effect of interferon alpha 2b. These two cystein groups even led to inconcistence of interferon alpha 2b activity due to interchangeable confirmation (data not published). Therefore, replacement of these two cystein groups revealed better biological effect as reported here as confirmed to native form.

## Conclusion

Interferon alpha 2b is a potent cytokine and widely used in clinic for antiviral infection and various cancers. In particular antitumor effect, this activity has been proposed to be of main pathway in mediating its antitumor effect. One of the first molecular effects on the cell cycle machinery following the addition of IFN is an upregulation of p21 levels which subsequently triggered secondary events such as upregulation of p27. Apoptotic pathway through activation of caspase-3 mediated by upregulation of STAT1 is also responsible for antitumor effect of IFNα2b. This phenomena was confirmed in this report using our developed interferon alpha 2b. The substitution of 2 cystein groups with aspartic acid does not diminish the antitumor effect, even better especially in the proapoptoc effect. This suggests that 2 cystein residues at position 2 and 99 are not responsible for biological activity of this protein. The better effect of substituting these cysteins seems to be relevant with the consistent conformation when 2 disulphide bridges are removed.

## Experimental

### Materials

Dulbecco’s modified eagles medium (DMEM, Gibco, USA), fetal bovine serum (Gibco, USA), penicillin and streptomycin (Sigma, USA), gentamycin (Sigma, USA), dimethylsulfoxide (Merck, Germany), trypsin (Merck, Germany), EDTA (Merck, Germany), Na_2_HPO_4_ (Merck, Germany), NaH_2_PO_4_ (Merck, Germany)_,_ isopropyl thiogalactopyranoside (IPTG, Sigma, USA) MTT (Merck, Germany), Tris base, (Merck, Germany), acetic acid (Merck, Germany), Ni TED column (Protino, USA), Kalferon (Kalbe farma, Indonesia), propidium iodide (Invitrogen, USA), MagaZorb® Total RNA Mini-Prep Kit (Promega, USA), RT-PCR kit (Promega, USA), PCR kit (Promega USA), primers : p27 (Forward and Reverse), casp3 (Forward and Reverse), GAPDH (Forward and Reverse) (First Base, Singapore), agarose (Boehringer Mannheim, Germany), DNA ladder 1 kpb (Fermentas, USA), sucrose (Sigma, USA), bromphenol blue (Sigma, USA) and ethidium bromida (Sigma, USA).

### Bacterial Strains, Plasmids and Cell culture

pET32b-*hifnα2b* recombinant plasmid from previous work was used as a template for SDM [8]. Luria Bertani (LB) broth containing 100 µg/mL of ampicillin was used for cultivation and LB containing 0.5 mM of IPTG was used as an inducer in gene expression step. Human hepatoma HepG2 cells were obtained from LIPI (Indonesian Institute for Science). The cells were cultured in DMEM medium (Sigma, USA) containing 10% fetal bovine serum (FBS) (Biochrome KG, Germany). Certain passage was used for this experiment.

### Site Directed Mutagenesis

As described previously by Ningrum et al [9] to do mutation, mutagenic primers were designed using DNA Star (DNA Star Inc, USA). First PCR-based SDM was applied to substitute TGT_2_ into GAT. Recombinant pET32b carrying hifnα2b ORF was used as a template. PCR was done in 50 μL reaction volume containing 20 mM dNTP, 1 x *Pfu* buffer, 2.5U of *Pfu* DNA polymerase, 150 ng of SDMPFORC2D primer, 150 ng of SDMPREVC2D primer, and 50 ng template. PCR product was treated by 10 U *Dpn*I at 37°C for 1 h and transformed into *E. coli* Top10. Recombinant pET32b that contained mutated hifn2b C2D ORF and pET32b carrying wildtype hifnα2b ORF were used as templates in second SDM step, which was substituting TGC_99_ into GAC. The PCR was performed as described previously. The mutagenic primers used were SDMPFORC99D and SDMREVC99D. The PCR was performed 12 cycles at 54°C for 1 min for annealing. PCR product was transformed into *E. coli* TOP10. Characterized recombinant plasmid was retransfromed into E. coli BL21(DE3) and used for further step.

### Protein Overproduction, Purification and Characterization

Overproduction of the proteins was performed using optimized condition reported previously [9]. The proteins were isolated as soluble and inclusion body (IB) forms. Soluble proteins were affinity purified by Nickel column according to manufacturer’s instruction (Protino, USA). All crudes and purified proteins (as soluble and IB) were analyzed using 15% Sodium Dodecyl Sulphate Polyacrylamide Gel Electrophoresis (SDS-PAGE) under denaturing condition and 10% PAGE under non denaturing condition for purified proteins. Protein concentration was determined using Bradford method based on coomassie blue staining.

### Cell cultivation

HepG2 cells were thawed and washed with 10 mL of DMEM medium containing antibiotic penicillin (100 units/mL), streptomycin (100 mg/mL), amphotericin B (0.025 mg/mL) and gentamycin (100 mg/mL). Cells were grown in the same medium containing 20% fetal bovine serum (FBS) at 37°C and 5% CO_2_. After reaching 90% confluency, cells were sub cultured in DMEM containing10% FBS. Cell washing was performed by using phosphate buffer saline (1.15 g Na2HPO4; 0.2 g KH2PO4; 8 g NaCl and 0.2 g KCl per liter, pH 7.2). The detachment was applied with 1 mL of trypsin-EDTA (0.25% trypsin in 0.53 mM EDTA) at 37°C for 5 min. After sub culture confluency reached 75%, the cells were washed, detached and subsequently transferred into a 96-well plate for further study.

### Antiproliferation assay

Prior to the experiment, optimization for MTT assay was carried out on three parameters, i.e: cell number, pretreatment condition and incubation time. Pre treatment condition was performed by comparing synchronized with unsynchonized condition. Synchronized was carried out by cell exposure to DMEM media only (without FBS) for 18 h. Commercial rhIFNα-2b was used as a standard. Optimum condition was used for further step on antiproliferation assay of proteins. Each time before using the proteins, their stability check was conducted in accordance with obtained optimum conditions. The stability was monitored by using non reducing SDS-PAGE. The electrophoregram band thickness was analyzed by ImageJ software (free downloaded from http://imagej.en.softonic.com). Treated cells in 96-well plate were washed twice with100 µL of PBS. 100 µL of DMEM medium containing 10% FBS and 20 µL of MTT (5 mg/mL) were added to each well. Cells were then incubated for 3 hours and the medium was discarded. Formazan crystals formed at the bottom of the well was dissolved in 200 µL of DMSO and then homogenized. Dissolved formazan was measured at wave lenght of 515 nm and the percentage of viable cell was compared to control (untreated cells). The experiments were done in triplicates.

### Tryphan blue staining

The media in each 96-well were removed and washed three times with PBS. The cells were detached by trypsin-EDTA for 5 min. Cell suspension was immediately diluted with medium. Staining was carried out by mixing cell suspension with trypan blue (1:1). The mixed suspension was incubated for 15 min at room temperature. 10 µL of stained cell was dripped on the hemocytometer counting chamber and analysed under microscope. The percentage of viable cell was compared to control (untreated cells). The experiments were done in triplicates.

### Cell cycle analysis

Treated cells in 24-well plate were washed with PBS and detached by trypsin-EDTA. The cell suspension in DMEM medium was centrifuged at 8000 g for 5 min and the supernatant was discarded. Cells were resuspended in100 µL of absolute ethanol and incubated at 4°C for 15 min. Cells were recentrifuged at same speed and resuspended in 250 µL of PBS and 50 µL of propidium iodide. Propidium iodide was used to stain the DNA of the cultured cells for the quantitative measurement of cellular DNA content by flow cytometry. Prior to the measurement, the cell suspension was incubated for 30 minutes in a dark room. Flow cytometry was performed on Beckman Coulter Epics XL-MCL (USA). Data were analysed for apoptosis and cell cycle using Multicycle Software. Apoptosis ratio of cultured HepG2 cells were measured as percentage of hipodiploidic peak.

### Gene expression analysis

Total RNA isolated from treated cells was used as a template. cDNA was synthesized from the same concentration of total RNA by using specific primers i.e. GAPDH, p27KIP, p21V1, p21B and caspase 3 (Table 1). cDNA amplification products were characterized by agarose gel electrophoresis and stained with ethidium bromide. The intensity of the bands was measured by ImageJ software to obtain semi-quantitative analysis. GAPDH band was used as internal control. Treated cells in 6-well plate were washed with PBS and detached with trypsin-EDTA. The cell suspension was centrifuged at 500 g and the supernatant was discarded. Total RNA was isolated from cells by using MagaZorb® Total RNA Mini-Prep Kit (Promega, USA) and the concentration was measured at 280 nm by spectrophotometer UV/Vis (Gene quant, USA). RT PCR was performed for each gene by RT-PCR kit (Promega, USA) at 0.5 µg of total RNA, 200 μM of dNTPs, 1.5 mM of MgCl_2_, 1U of *Taq* DNA polymerase and 2.5 µM of primer. cDNA amplification was carried out for each gene (Table 2).

### Study the effect of rIFNα2b on pSTAT 1 regulation

The protein expression of pSTAT1 after rhIFNα2b treatment was also analyzed separately *in vitro* using HepG2 cells. Cells were seeded in 12-well culture plates (1.5×10^5^ cells per well) in Dulbecco’s modified Eagle’s medium (DMEM) supplemented with 10% FBS and antibiotics (100 U/mL penicillin and 100 ng/mL streptomycin) and grown for two days. Cells were then incubated with medium and commercial rhIFNα2b (Jena Bioscience, Germany) 100 ng/mL containing medium as negative and positive controls; and various concentrations of rhIFNα2b native and mutein containing medium for 4h. The cells were lysed in loading buffer (0.5 mM Tris-HCl pH 6.8, Glycerol, 8% SDS, 400 mM DTT, and bromophenol blue) on ice and sonicated for 5 sec. The cell lysate from each sample was applied on the SDS-Page gel (10%) and transferred to PVDF membranes. The membrane was blocked for 1h in TBST containing 5% non-fat dried milk and was incubated with anti-Phospo-Stat1 (Tyr701) (1:1000, Cell Signaling Technology Inc., Beverly, MA) and anti-GAPDH (1:10000, SIGMA) for overnight at 4 °C. The membrane was then washed with TBST and incubated for 1h at room temperature with a secondary horseradish peroxidase-coupled IgG antibody (DAKO). The membrane was washed for three times with TBS and protein bands were visualized using ECL detection reagent.

### Statistical analyses

All data were analyzed with SPSS program. Statistical differences between the control and IFN treatment groups were calculated by unpaired student’s t-test and considered significant at P < 0.05.
